# P-413. Duration of Antibiotic Prophylaxis in Children with Anatomic or Functional Asplenia: A Multidisciplinary, Multicenter Survey

**DOI:** 10.1093/ofid/ofaf695.630

**Published:** 2026-01-11

**Authors:** Kathryn E Weakley, David Foley, Marylou M Dryer, Kerry McGowan, Victoria A Statler

**Affiliations:** Norton Children's and University of Louisville School of Medicine, Louisville, KY; University of Louisville School of Medicine, Louisville, Kentucky; Norton Children's Heart Institute, Loouisville, Kentucky; Norton Children's Hospital/University of Louisville, Louisville, Kentucky; Norton Children's and University of Louisville School of Medicine, Louisville, KY

## Abstract

**Background:**

There are no U.S. guidelines outlining the appropriate duration of antibiotic prophylaxis for patients with anatomic or functional asplenia.

**Figure:**
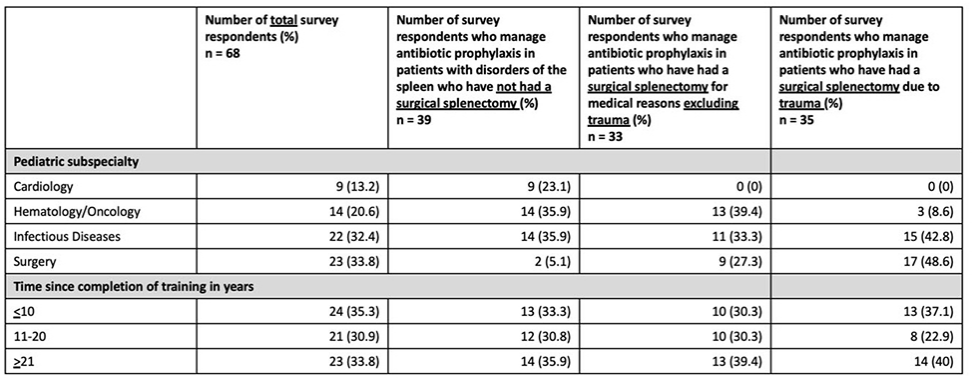
Demographics

**Figure:**
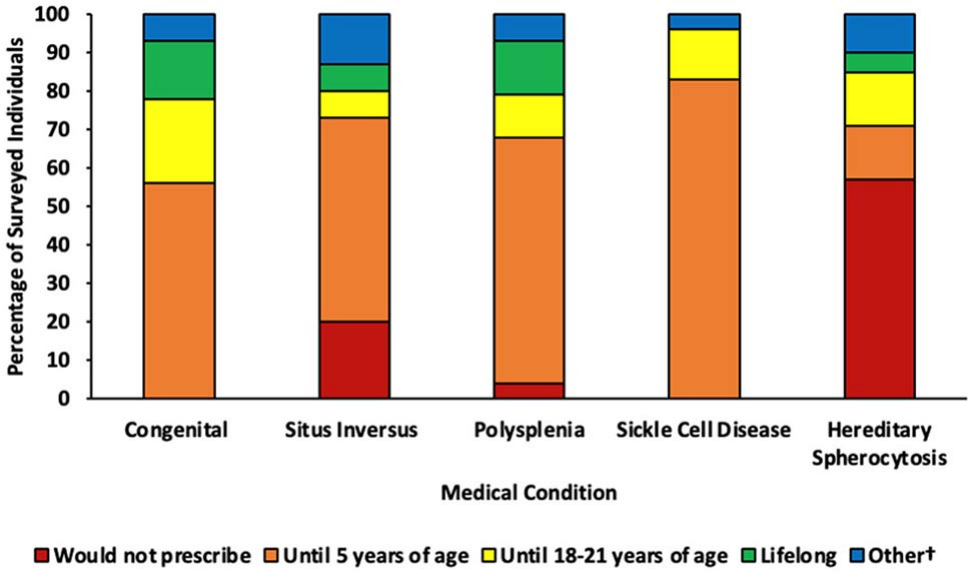
Duration of prophylaxis in patients with disorders of the spleen who have not undergone a surgical splenectomy †Duration dependent on splenic function testing; Minimum of 5 years but potentially lifelong with shared decision making

**Methods:**

An online survey was sent to U.S. pediatric infectious diseases (ID), hematology/oncology (H/O), cardiology, and surgery subspecialists. Participants were asked about their prescribing practices in patients with asplenia. Descriptive analysis was performed.

**Figure:**
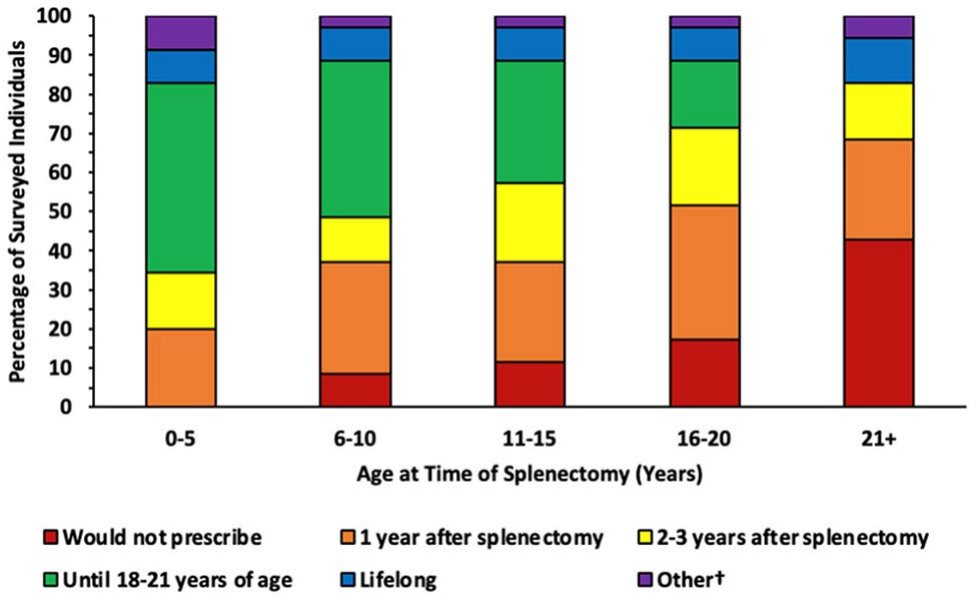
Duration of prophylaxis in patients who underwent a surgical splenectomy due to trauma †1 year after splenectomy or until 5 years of age, whichever is longer; Minimum of 1 year after splenectomy or until 5 years of age, but potentially lifelong with shared decision making; Do not manage patients >21 years of age

**Figure:**
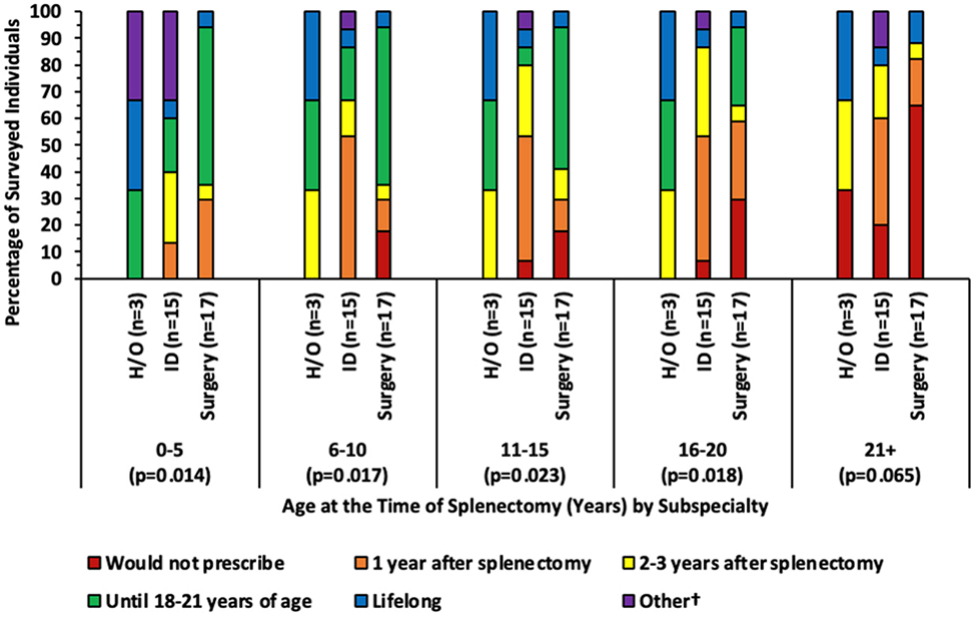
Duration of prophylaxis in patients who underwent a surgical splenectomy due to trauma according to subspecialty †1 year after splenectomy or until 5 years of age, whichever is longer; Minimum of 1 year after splenectomy or until 5 years of age, but potentially lifelong with shared decision making; Do not manage patients >21 years of age

**Results:**

There were 68 respondents; 34% surgery, 32% ID, 21% H/O, and 13% cardiology. Approximately 1/3 had been practicing for < 10 yrs, 1/3 for 11-20 yrs, and 1/3 for > 21 yrs (Table 1). 39 respondents managed prophylaxis in patients with non-surgical disorders of the spleen; most prescribed prophylaxis until 5 years of age for patients with sickle cell disease, but duration varied for patients with congenital anatomic asplenia, situs inversus, polysplenia, and hereditary spherocytosis (Figure 1). 33 respondents managed prophylaxis in patients who underwent a surgical splenectomy due to medical conditions excluding trauma, and 35 prescribed for patients who underwent a surgical splenectomy due to trauma. Duration of prophylaxis decreased with older age at time of splenectomy (Figure 2). Prescribing practices differed according to subspecialty for patients who underwent surgical splenectomy due to trauma (Figure 3) and for patients with hereditary spherocytosis. Prescribing practices differed according to time since completion of training for patients with situs inversus. Across all categories, 78% would likely extend duration of prophylaxis in incompletely vaccinated patients, and 94% would likely restart prophylaxis if a patient developed invasive pneumococcal disease after stopping prophylaxis. 40 institutions were represented; 20 institutions had 2 or more respondents. There was practice variation among individuals from the same institution. Only 31% of respondents had institutional guidelines for asplenia. 93% reported their clinical practice would benefit from consensus guidance.

**Conclusion:**

There is significant practice variation in the duration of antibiotic prophylaxis prescribed for patients with asplenia. These results illustrate the need for outcomes research and expert consensus guidance.

**Disclosures:**

Kathryn E. Weakley, MD, MSc, BioNTech: Grant/Research Support|Merck Sharp & Dohme LLC: Grant/Research Support|Pfizer: Grant/Research Support Victoria A. Statler, MD, M.Sc., Merck: Grant/Research Support|Sanofi: Advisor/Consultant|Seqirus: Advisor/Consultant

